# DNA Barcoding of the Palaearctic Elfin Butterflies (Lepidoptera, Lycaenidae) with a Description of Four New Species from Vietnam †

**DOI:** 10.3390/insects14040352

**Published:** 2023-04-02

**Authors:** Anatoly Krupitsky, Nazar Shapoval, Galina Shapoval

**Affiliations:** 1 Department of Entomology, Biological Faculty, Lomonosov Moscow State University, Leninskie Gory, GSP-1, korp. 12, 119991 Moscow, Russia; 2 Severtsov Institute of Ecology and Evolution, Russian Academy of Sciences, Leninsky Prospect 33, 119071 Moscow, Russia; 3 Department of Karyosystematics, Zoological Institute, Russian Academy of Sciences, Universitetskaya nab. 1, 199034 Saint-Petersburg, Russia; nazaret@bk.ru (N.S.); galinakuftina@mail.ru (G.S.)

**Keywords:** Theclinae, Eumaeini, morphology, genitalia, genetic divergence, DNA barcoding, phylogenetic analysis

## Abstract

**Simple Summary:**

We studied the Palaearctic elfin butterflies, a group of hairstreaks (family Lycaenidae, subfamily Theclinae, tribe Eumaeini) comprising about 50 species belonging to three genera, *Ahlbergia* Bryk, 1947, *Cissatsuma* Johnson, 1992, and *Novosatsuma* Johnson, 1992, known mostly from China. Members of this group are still underexplored, and the taxonomy of the Palaearctic elfin butterflies is complicated both at the species and the generic level. We performed an analysis based on a 658 bp region of the *COI* mitochondrial gene, covering the previously considered genera *Ahlbergia*, *Cissatsuma*, *Novosatsuma* and *Callophrys* Billberg, 1820, in order to properly discriminate the Palaearctic elfin butterfly species and reveal their phylogenetic positions. The phylogenetic analysis revealed Holarctic *Callophrys* sensu lato as a strongly supported monophyletic group, but none of the usually treated Palaearctic elfin butterfly genera was recovered as a monophyletic unit. Additionally, we revealed and described four new species of Palaearctic elfin butterflies from Northeast Vietnam.

**Abstract:**

Phylogenetic analysis is provided for the first time for 12 species of Palaearctic elfin butterflies, members of the previously recognized genera *Ahlbergia* Bryk, 1947, *Cissatsuma* Johnson, 1992, and *Novosatsuma* Johnson, 1992, based on the barcoding region of the mitochondrial *cytochrome C oxidase subunit I* gene (*COI*). Comparison of the *COI* barcodes revealed very low levels of genetic divergence between the species of the Palaearctic elfin butterflies and *Callophrys* Billberg, 1820 sensu stricto. *COI*-based phylogeny revealed that Palaearctic *Callophrys* and the Palaearctic elfin butterflies, except *Cissatsuma*, are polyphyletic. Four new sympatric species, namely, *Callophrys* (*Ahlbergia*) *hmong* sp. n., *C.* (*A.*) *tay* sp. n., *Callophrys* (*Cissatsuma*) *devyatkini* sp. n. and *C.* (*A.*) *dao* sp. n. are described from Ha Giang Province, North Vietnam, based on wing colouration, the morphologies of the male and female genitalia, and differences in *COI* sequences. Discovery of the new species expands the distribution range of the group towards the southeast, beyond the Palaearctic region.

## 1. Introduction

The term “Palaearctic elfin butterflies” is traditionally used for three genera of the Eumaeini hairstreaks, *Ahlbergia* Bryk, 1947, *Cissatsuma* Johnson, 1992, and *Novosatsuma* Johnson, 1992, inhabiting mountains of East Asia, with most species in China, mainly in the provinces of Sichuan and Yunnan. Consistent exploration of the Palaearctic elfin butterflies was started by Johnson [1], who outlined these genera on the basis of external and genitalic characters and described several new species and subspecies. Since Johnson’s study, a number of new species have been described [2,3,4,5,6,7,8,9,10,11,12,13,14,15,16], and several species have been synonymised or transferred from one genus to another [11,12], so that the total number of Palaearctic elfin butterfly species at present is 46.

The group in question presents one of the most challenging tasks in the taxonomy of the Palaearctic butterflies, both at the species and genus levels.

At the species level, the taxonomy of the Palaearctic elfin butterflies is complicated by the following points: (1) species of the Palaearctic elfin butterflies are very rarely found in collections, often being known only from century-old type specimens; (2) some taxa were previously known only from either males or females, e.g., in Johnson’s review [1], different sexes of conspecific specimens were not associated and were described as distinct species under different genera, as demonstrated by Huang and Zhou [11].

At the generic level, the taxonomy of the Palaearctic elfin butterflies is not well understood yet. While the Nearctic elfin butterflies are usually considered as several subgenera within the diverse Holarctic genus *Callophrys* Billberg, 1820 [17,18], there is still no consensus among specialists about whether *Ahlbergia*, *Cissatsuma* and *Novosatsuma* represent separate genera, subgenera of *Callophrys* or synonyms of the latter. In the recent morphology-based studies, these taxa are usually considered as genera [13,15,16], but with some amendments, as the genera *Ahlbergia* and *Novosatsuma* sensu Johnson [1] lack the unambiguous morphological diagnostic characters in the male and female genitalia [11]. However, a few molecular phylogenetic studies involving the Palaearctic elfin butterflies support the opposite point of view. The phylogenetic analysis of a fragment of the mitochondrial *cytochrome C oxidase subunit I* gene (*COI*), the barcoding region [19], resulted in a topology in which *Ahlbergia* and *Cissatsuma* are nested within the clade of the Palaearctic *Callophrys*. In the multilocus molecular phylogenetic analysis of the whole tribe Eumaeini [20], *Ahlbergia* is considered a synonym of *Callophrys*. In the genomics-based phylogenetic analysis of Eumaeini genera by Robbins and co-authors [21], *Ahlbergia*, *Cissatsuma* and *Novosatsuma* are considered synonyms of *Callophrys*. It is worth noting that the latter phylogenetic studies included limited numbers of species of the Palaearctic elfin butterflies. The recent study of mitochondrial (*COI*) and nuclear (*ribosomal protein S5* (*RpS5*), *wingless* (*Wg*) and *sarco/endoplasmic reticulum calcium ATPase* (*Ca-ATPase*)) genes of *Callophrys rubi* (Linnaeus, 1758) and *Ahlbergia frivaldszkyi* (Lederer, 1853) revealed a very low level of genetic differentiation between them and confirmed that these species occasionally hybridise in nature [22].

The above-mentioned points clearly indicate that a review of the Palaearctic elfin butterflies and *Callophrys* based on a molecular phylogenetic analysis combined with studies of morphology is needed to delimit species and genera within this group and compile clear morphological diagnoses.

The distribution and biodiversity of the Palaearctic elfin butterflies are not clearly explored either. Although *Ahlbergia*, *Cissatsuma* and *Novosatsuma* are characterised by mostly Palaearctic distribution and known to be temperate mountain insects, several species of these genera venture into Indochina and inhabit mountains of Assam and Nagaland in India, Myanmar, Thailand and Laos [14,23,24,25,26].

The butterfly fauna of Vietnam is studied rather well [27,28,29], but data on Vietnamese elfin butterflies are nearly absent in publications, in spite of numerous records of these genera from the neighbouring mountains of Laos and China. Only one record has been published: *Ahlbergia chalcidis* Chou & Li, 1994, which was mentioned in a study devoted to butterflies of the Dong Van karst plateau in Ha Giang Province, Northern Vietnam [30]. Additionally, four specimens identified as *Novosatsuma pratti* (Leech, 1889) collected in Ha Giang were mentioned in the list of specimens from the Suguru Igarashi collection [31].

Our study of elfin butterflies collected in Ha Giang Province, North Vietnam, from 2003 to 2021 revealed four undescribed species. In order to properly discriminate the new species and reveal their phylogenetic positions, we used an integrative taxonomic approach combining a *COI* barcoding-based molecular phylogenetic analysis and an analysis of morphological characters.

In this paper, we (1) provide for the first time a hypothesis of the phylogeny of the Palaearctic elfin butterflies based on a 658 bp region of the *COI* mitochondrial gene, covering the previously distinguished genera *Ahlbergia*, *Cissatsuma*, *Novosatsuma* and *Callophrys*; (2) describe the revealed new species on the basis of *COI* barcoding data and analysis of morphology; and (3) discuss the taxonomy of the Palaearctic elfin butterflies.

## 2. Materials and Methods

### 2.1. Nomenclature Used in This Study

In the present work, we follow the concept of a diverse Holarctic genus *Callophrys* [17,18,19] and for taxonomic purposes tentatively treat *Callophrys* sensu stricto, *Ahlbergia*, *Cissatsuma* and *Novosatsuma* as subgenera of the genus *Callophrys*, leaving all the combinations previously used within this group [13] at subgeneric level to avoid taxonomic confusions and to make a framework for future studies.

### 2.2. Sampling, DNA Extraction, PCR and Sequencing

Our ingroup dataset included 12 species of all previously recognised genera of the Palaearctic elfin butterflies, namely, *Ahlbergia*, *Novosatsuma* and *Cissatsuma.* Eleven of them (37 specimens) were sequenced during this study (Table 1), and three *COI* sequences (OL457026, MW785858 and MW785859) of *Callophrys* (*Ahlbergia*) *frivaldszkyi* were obtained from GenBank.

Additionally, the *COI* sequences of the genus *Callophrys* (sensu lato) available from GenBank were included in the phylogenetic analysis: two Palaearctic *Callophrys* species, namely, *Callophrys* (*Callophrys*) *rubi* and *C.* (*Callophrys*) *avis* Chapman, 1909, and eight Nearctic species, namely, *Callophrys* (*Cisincisalia*) *johnsoni* (Skinner, 1904), *C*. (*Incisalia*) *augustinus* (Westwood, 1852), *C*. (*I.*) *henrici* (Grote & Robinson, 1819), *C*. (*I.*) *eryphon* (Bosiduval, 1852), *C*. (*I.*) *niphon* (Hübner, 1823), *C*. (*I.*) *irus* (Godart, 1824), *C*. (*I.*) *polios* (Cook & Watson, 1907) and *C*. (*Mitoura*) *gryneus* (Hübner, 1819). The *COI* sequence of the hairstreak *Neolycaena* (*Rhymnaria*) *baidula* Zhdanko, 2000 was used as the outgroup. Thus, the final dataset for the phylogenetic analysis included 53 specimens.

One leg of each specimen was taken for DNA extraction using a QIAamp DNA Investigator Kit (Qiagen, Venlo, The Netherlands), following the manufacturer’s protocol. The mitochondrial DNA barcode (a 658 bp fragment of the *COI* gene) was amplified using LCO1490/HCO2198 [32] and LepF/LepR primer pairs [33]. In case standard lepidopteran barcode primers failed to yield a sufficient product, we amplified full-length barcode fragments using the primer pair combinations LepF/MH-MR1 + MH-MF1/LepR and LCO1490/MH-MR1 + MH-MF1/HCO2198 [34]. For specimens collected a long time ago, self-designed primer pairs, amplifying six short overlapping fragments, were used to obtain targeted fragments. The primers used in this study are listed in Table 2.

The PCR amplifications were performed in a 25 μL reaction volume per sample. Each reaction contained 1 μL template DNA (ca. 10–50 ng genomic DNA, measured with a NanoDrop Lite spectrophotometer, Thermo Fisher Scientifics, Waltham, MA, USA), 1.3 μL of both forward and reverse primers aliquoted to a standard concentration of 10 μM, 5 μL of 5× ScreenMix (Evrogen, Russia, Moscow), and 16.4 μL of ddH2O. The temperature profile was as follows: initial denaturation at 95 °C for 5 min, followed by 35 cycles of denaturation at 94 °C for 30 s, annealing at 50 °C for 30 s and extension at 72 °C for 1 min 30 s, with a final extension at 72 °C for 10 min. The purified PCR products were subjected to further sequencing. Sequencing of the double-stranded product was carried out at the Research Resource Center for Molecular and Cell Technologies (St. Petersburg State University, St. Petersburg, Russia) using an ABI 3500xL analyser (Applied Biosystems, Waltham, MA, USA). All sequences obtained in this study were deposited in GenBank (http://www.ncbi.nlm.nih.gov/, accessed on 29 March 2023).

### 2.3. Sequence Processing and Phylogenetic Analysis

The sequences were aligned with the MUSCLE algorithm implemented in GENEIOUS v.7.1.9 [35]. Phylogenetic analyses were performed using the Bayesian inference (BI) approach. The Bayesian estimation of posterior probability was performed in MrBayes v.3.2.5 [36], applying the GTR + G + I evolutionary model, as suggested by PartitionFinder v.2.1.1 [37]. Markov chains were sampled at intervals of 500 generations. Two runs of ten million generations with four chains (one cold and three heated) were performed. The first 25% of sampled trees were discarded as burn-in. We regarded tree nodes with BI posterior probabilities (PPs) > 0.95 to be sufficiently resolved a priori. The final phylogenetic tree images were rendered in FigTree v.1.4.0 (http://tree.bio.ed.ac.uk/software/figtree/, accessed on 20 November 2022) and then edited using Adobe Illustrator CC 2018 and Adobe Photoshop CC 2014.2.2 software. Minimal uncorrected *COI p*-distances were calculated using MEGA7 [38].

### 2.4. Morphology and Distribution

Morphological characters of 44 specimens of the Palaearctic elfin butterflies representing 12 species previously delimitated on the basis of morphology (including four new ones and one of uncertain status, *C*. (*A*.) sp.) of the previously recognised genera *Ahlbergia*, *Cissatsuma* and *Novosatsuma* are studied within this work.

The characters used for the species delimitation of the Palaearctic elfin butterflies were selected following Johnson [1], Huang and Zhou [11] and Huang and Zhu [13]. The nomenclature for the genitalia and wing patterns was adapted after Johnson [1] and Krupitsky [15]. The nervuration nomenclature follows the Comstock–Needham system adapted for butterflies [39]. For the examination of the male genitalia, the abdomens of the studied specimens were removed and macerated in 10% KOH. After cleaning in water and dehydration in 96% EtOH, a genital capsule with valvae and separated aedeagus were placed in a drop of glycerol, covered with a cover glass and photographed. In the case of the genital capsule, photos were taken in ventral and lateral views, and in lateral view in the case of the aedeagus.

The images of the studied specimens were taken with a Canon EOS 5D mark II digital camera (Canon Inc., Tokyo, Japan) equipped with a Sigma 150 mm f2.8 lens (Sigma Corporation, Kawasaki, Japan), using an originally developed light system and a Canon Speedlight 430 EX flash (Canon Inc., Tokyo, Japan) with a diffuser. The images of the genitalia were taken with a Canon EOS 6D digital camera (Canon Inc., Tokyo, Japan) equipped with a Canon MP-E 65 mm f/2.8 lens (Canon Inc., Tokyo, Japan), using two Micromed Dual Goose illuminators (Micromed, St. Petersburg, Russia). Obtained images were edited using Adobe Photoshop CC 2014.2.2 software.

The distribution map was generated using SimpleMappr (http://www.simplemappr.net, accessed on 31 August 2022) on the basis of the literature [12,13,14,23,40] and label data for the studied specimens and was edited using Adobe Photoshop CC 2014.2.2 software.

The studied specimens are deposited in the collection of the Department of Entomology, Moscow State University (CEDM); the private collection of Vasily Tuzov, Moscow, Russia (CVTM); the collection of the Zoological Institute, the Russian Academy of Sciences, St. Petersburg, Russia (ZISP); and the collection of the Zoological Museum of Moscow State University, Moscow, Russia (ZMMU).

## 3. Results

### 3.1. Phylogenetic Relationships

**Figure 1:**
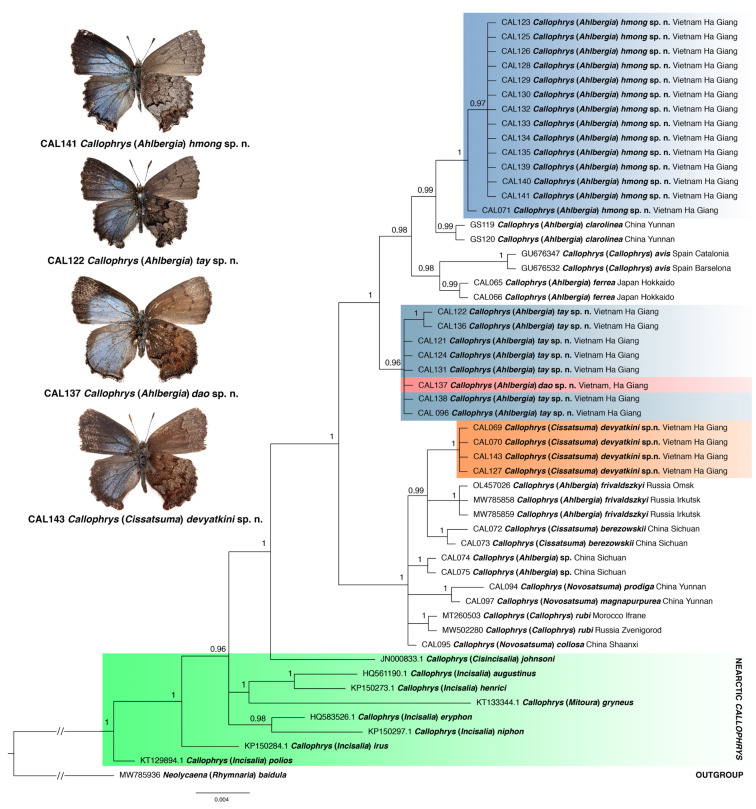
The Bayesian tree of *Callophrys* sensu lato based on analysis of the *cytochrome oxidase subunit I* (*COI*) gene fragment. Numbers at nodes indicate Bayesian posterior probabilities (PPs). Scale bar = 0.004 substitutions per position.

The phylogenetic analysis revealed Holarctic *Callophrys* sensu lato as a strongly supported monophyletic group (Figure 1). The Palaearctic clade comprising elfin butterflies and *Callophrys* sensu stricto was also recovered with high support, with *C*. (*Cisincisalia*) *johnsoni* as a sister species. Within the Palaearctic clade, all recovered lineages received PPs ≥ 0.96. The genera *Callophrys*, *Ahlbergia* and *Novosatsuma* were recognised as polyphyletic groups.

Two deep-branching lineages within the Palaearctic clade were recovered on the phylogenetic tree. The first lineage comprises *Callophrys avis* and four *Ahlbergia* species, including the type species of the genus, *A. ferrea* (Butler, 1866). Three new species tentatively classified as members of the subgenus *Ahlbergia* based on the analysis of morphology belong to this clade. One of them (described below as *C*. (*A*.) *hmong* sp. n.) is a sister species to *C*. (*A*.) *clarolinea* (Huang and Chen, 2006), with a sister clade of them both comprising *C*. (*C*.) *avis* and *C*. (*A*.) *ferrea*. The second clade uniting a new species described below as *C*. (*A*.) *tay* sp. n. and a new species described below as *C*. (*A*.) *dao* sp. n. is sister to the clade uniting the former and the latter clades.

The second deep-branching lineage comprises *Callophrys* (*Callophrys*) *rubi* (the type species of the genus) and members of *Ahlbergia*, *Cissatsuma* and *Novosatsuma*. Within this clade, four monophyletic entities are recognised: (1) the clade comprising the new species described below as *C*. (*Cissatsuma*) *devyatkini* sp. n., *C*. (*Cissatsuma*) *berezowskii* (Krupitsky, 2018) and *C*. (*A*.) *frivaldszkyi*; (2) the clades of *C*. (*A*.) sp. and *C*. (*C*.) *rubi*; and (3) the clade uniting *C*. (*Novosatsuma*) *prodiga* (Johnson, 1992) and *C*. (*N.*) *magnapurpurea* (Johnson, 1992). All these clades appear on an unresolved branch together with a single specimen of *C*. (*N.*) *collosa* (Johnson, 1992).

Among the 44 specimens belonging to 14 species (including *C*. (*C*.) *rubi* and *C*. (*C*.) *avis*), mean uncorrected pairwise distances (*p*-distances) range from 0.0% (between *C*. (*A*.) *tay* sp. n. and *C*. (*A*.) *dao* sp. n.) to 2.72% ± 0.64% (between *C*. (*A*.) *hmong* sp. n. and *C*. (*N.*) *prodiga*) (Table 3).

### 3.2. Taxonomy

#### 3.2.1. *Callophrys* (*Ahlbergia*) *hmong* Krupitsky, Shapoval & Shapoval, sp. n. (Figure 2A,B, Figure 3A and Figure 4A)

LSID urn:lsid:zoobank.org:act:C5478CD5-567E-4F05-BF6C-AEB34019272B.

**Figure 2:**
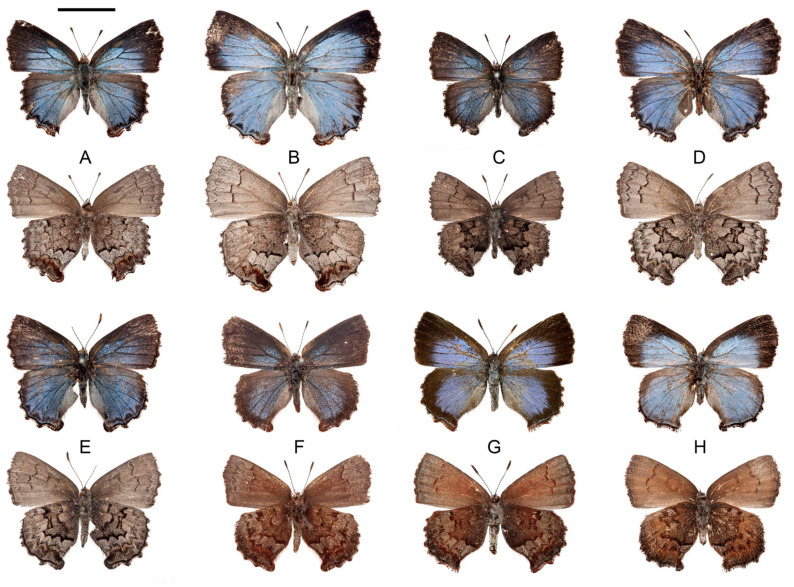
Habitus of the new species. Scale bar equals 10.0 mm. (**A**) *Callophrys* (*Ahlbergia*) *hmong* Krupitsky, Shapoval & Shapoval, sp. n., holotype, male, specimen code CAL141. (**B**) Id., paratype, female, specimen code CAL128. (**C**) *Callophrys* (*Ahlbergia*) *tay* Krupitsky, Shapoval & Shapoval, sp. n., holotype, male, specimen code CAL122. (**D**) Id., paratype, female, specimen code CAL121. (**E**) Id., paratype, male, specimen code CAL124. (**F**) *Callophrys* (*Cissatsuma*) *devyatkini* Krupitsky, Shapoval & Shapoval, sp. n., paratype, male, specimen code CAL143. (**G**) Id., holotype, female, specimen code CAL069. (**H**) *Callophrys* (*Ahlbergia*) *dao* Krupitsky, Shapoval & Shapoval, sp. n., holotype, female, specimen code CAL137.

**Figure 3:**
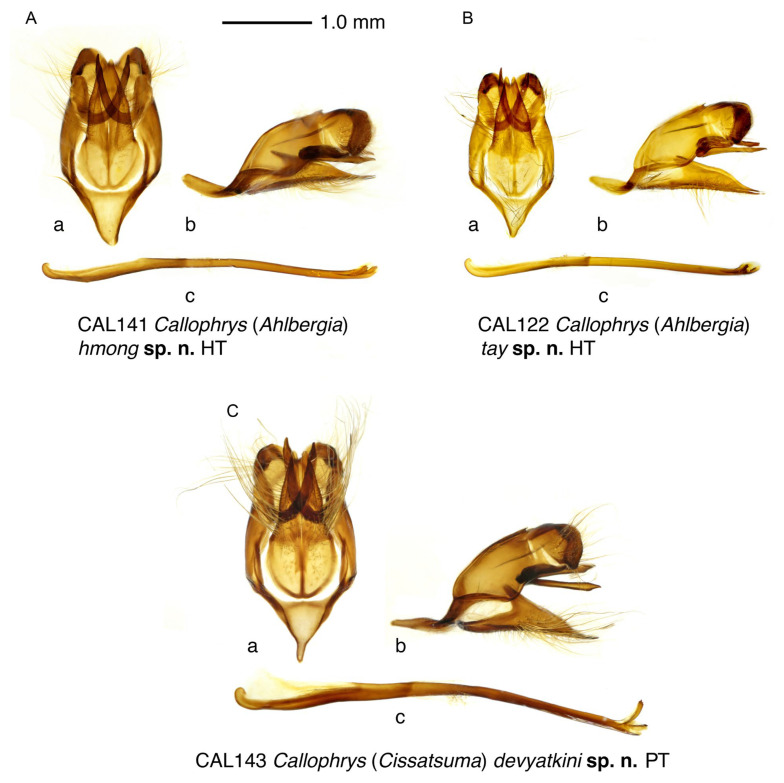
Male genitalia of the new species. (**A**) *Callophrys* (*Ahlbergia*) *hmong* Krupitsky, Shapoval & Shapoval, sp. n., holotype, specimen code CAL141. (**B**) *Callophrys* (*Ahlbergia*) *tay* Krupitsky, Shapoval & Shapoval, sp. n., holotype, specimen code CAL122. (**C**) *Callophrys* (*Cissatsuma*) *devyatkini* Krupitsky, Shapoval & Shapoval, sp. n., paratype, specimen code CAL143. **a**, genital capsule, ventral view; **b**, id., lateral view; **c**, aedeagus, lateral view.

**Figure 4:**
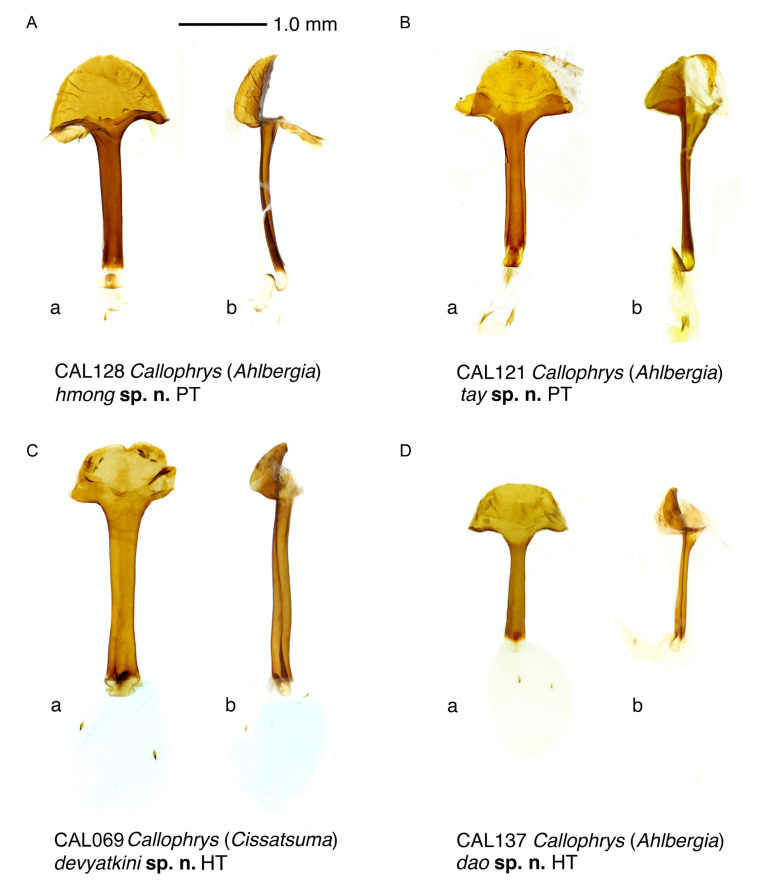
Female genitalia of the new species. (**A**) *Callophrys* (*Ahlbergia*) *hmong* Krupitsky, Shapoval & Shapoval, sp. n., paratype, specimen code CAL128. (**B**) *Callophrys* (*Ahlbergia*) *tay* Krupitsky, Shapoval & Shapoval, sp. n., paratype, specimen code CAL121. (**C**) *Callophrys* (*Cissatsuma*) *devyatkini* Krupitsky, Shapoval & Shapoval, sp. n., holotype, specimen code CAL069. (**D**) *Callophrys* (*Ahlbergia*) *dao* Krupitsky, Shapoval & Shapoval, sp. n., holotype, specimen code CAL137. **a**, ventral view; **b**, lateral view.

Type material. Holotype: ♂, specimen code CAL141, GenBank accession number OM630550, North Vietnam, Ha Giang Province, Dong Van District, Dong Van Karst Plateau, Sa Phin commune, 1400–1600 m a.s.l., 23°15′11″ N 105°15′01″ E, April 2021, local collector leg. (ZMMU). Paratypes: 13 ♂ (CAL123, OM630538; CAL125, OM630539; CAL126, OM630540; CAL129, OM630542; CAL130, OM630543; CAL132, OM630544; CAL133, OM630545; CAL134, OM630546; CAL135, OM630547; CAL139, OM630548; CAL140, OM630549; CAL147), ♀ (CAL128, OM630541), same data as holotype (CEDM); ♂, CAL148, same data as holotype (ZISP); ♂ (CAL071, OM630537), same locality, May 2018, local collector leg. (CEDM).

##### Diagnosis

*Callophrys* (*Ahlbergia*) *hmong* sp. n. differs from all known elfin butterflies in the combination of celestial-blue colouration of the dorsal side of the wings and greyish ventral side with broad whitish band and rusty lunules in spaces CuA1–2A. From the genetically closest species involved in our phylogenetic analysis, *C.* (*A.*) *clarolinea*, genetic divergence at the *COI* barcodes is 0.6% ± 0.29%, the new species differs in the light ventral side of both wings and rusty-brown lunules in spaces CuA1–2A (vs. darkened ventral side of the wings and dark-brown lunules in spaces CuA1–2A in *C.* (*A.*) *clarolinea*, cf. Figures 18–20 and 63–66 in [13]). From the somewhat similar species *C.* (*A.*) *bijieensis* (Huang & Sun, 2016), comb. nov., recently described from Guizhou Province, China, the new species differs with respect to the light basal disc (darkened in *C.* (*A.*) *bijieensis*, cf. Figures 16–20 in [12]), broader uncus and longer valva in the male genitalia (uncus narrower and valva shorter in *C.* (*A.*) *bijieensis*, cf. Figure 32 in [12]), and broader lamella postvaginalis, smaller junctures of lamellae and smaller signum with poorly developed small spines (vs. lamella postvaginalis narrower, junctures of lamellae larger; signum larger with two spines of unequal size in *C.* (*A.*) *bijieensis*, cf. Figure 39 in [12]).

**Figure 5:**
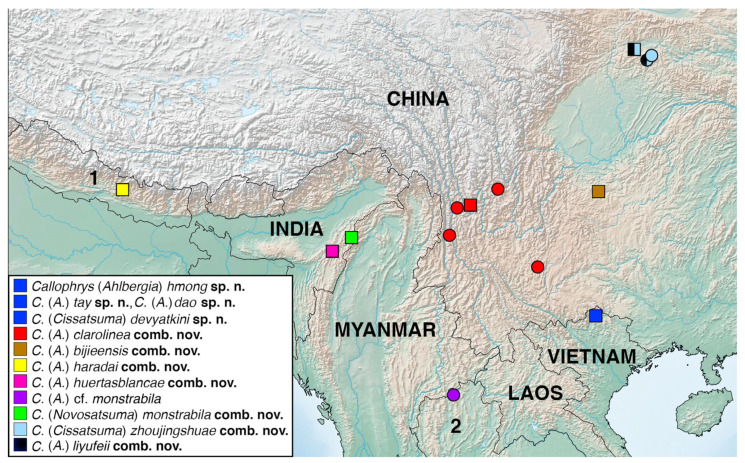
Distribution of the studied species of the elfin butterflies. Type localities are marked by squares. 1, Nepal; 2, Thailand.

Another superficially similar species, *C.* (*A.*) *huertasblancae* (Yoshino, 2016), comb. nov., was described from Nagaland, Arunachal Pradesh, Northeastern India. *C.* (*A.*) *hmong* sp. n. differs from the latter in celestial-blue colouration of dorsal side of wings in both sexes (vs. deeper blue in *C.* (*A.*) *huertasblancae*, cf. Figures 3, 5 and 7 in [14]), less contrasting beige disc and whitish postdiscal band of ventral side of hindwing (vs. contrasted grey disc and greyish postdiscal band in *C.* (*A.*) *huertasblancae*, cf. Figures 4 and 6 in [14]), rusty-brown lunules in spaces CuA1–2A (brown lunules in *C.* (*A.*) *huertasblancae*, cf. Figures 4 and 6 in [14]), and rather long narrow male genitalia with distal part of the valva with sides parallel and then gradually tapering to apex (vs. very short and broad male genitalia with distal part of valva gradually tapering to apex, cf. Figure 8 in [14]).

Additionally, *C.* (*A.*) *hmong* sp. n. is somewhat similar to *C.* (*A.*) *haradai* Igarashi, 1973, comb. nov., known from the holotype “female” (male, in fact, as evidenced by the androconial spot and the shape of the abdomen depicted in the photo included with the original description) from Nepal [40] and a couple of photos in nature [41]. Based on limited diagnostic information from the mentioned images of *C.* (*A.*) *haradai*, it can be concluded that the new species differs from the latter in the less developed blue field of the male forewing limited by the postdiscal area (vs. blue field reaching the submarginal area in *C.* (*A.*) *haradai*, cf. Figure 1 in [40]) and the less contrasted beige disc (vs. darkened disc in *C.* (*A.*) *haradai*, cf. Figure 2 in [40]).

##### Description

Male (Figure 2A). Head: antenna brown, white-ringed at bases of segments, antennal club brown; eye surrounded with white stripe, light brown with very short sparse hairs; frons with brown hairs, top of head with brown scales and hairs; palpus: palpomere 2 covered with whitish hairs and scales, palpomere 3 black.

Thorax: dark brown with bluish-grey hairs dorsally, densely covered with brown and rusty-brown hairs ventrally; femur dark brown, tibia and tarsus whitish with dark-brown stripes.

Abdomen: dark brown with whitish hairs dorsally, whitish ventrally, tip brown.

Forewing: triangular, with rounded apex and termen. Forewing length 14.0 mm in holotype, 11.0–15.0 mm in paratypes. Dorsal side black, cell and discal area in spaces M3–2A densely covered with celestial-blue scales; veins dark brown; base of costal area with suffusion of rusty-brown scales; outer margin same as background; fringe wavy, convex at veins, dark brown with admixture of beige scales; basal area of wing and inner margin covered with rather long whitish hairs, tornus with long dark-brown scales; androconial spot leaf-like, dark brown to beige, length about 2.0 mm. Ventral side brown, densely covered with whitish scales, base and costa with rusty-brown scales; postmedial line wavy, dark brown with white scales proximally and rusty-brown scales distally; spaces R3–CuA1 with crescent line consisting of dark-brown elements with groups of whitish scales distally; submarginal area with brown scales; outer margin brown; fringe as on dorsal side.

Hindwing: costa straight, with basal lobe, apex rounded, termen wavy; anal lobe well-developed, rounded. Dorsal side black with broad celestial-blue field in spaces Rs–CuA2 extending to submarginal area in spaces M3–CuA2, with V-shaped black incisions separating it from margin; veins black; spaces 2A–3A greyish blue; outer margin black, bordered by blue scales, more intensively in spaces M3–CuA2, and with whitish scales in half of space CuA2 and 2A near anal lobe; basal half of wing and inner margin covered with long sparse whitish hairs; fringe dark brown proximally, whitish distally, with long dark-brown scales at veins; anal lobe with long rusty-brown scales. Ventral side with brown base, postbasal marks dark brown with whitish scales proximally, developed in space Sc + R1 and cell, connected with marginal band of disc in space CuA2; marginal band of disc consisting of dark-brown broken lines, bold in spaces Sc + R1, Rs, CuA2–3A, with projection in space M3; space between postbasal marks and marginal band of disc covered with greyish scales; crescent line black, developed in spaces M3–2A, traced in other spaces; limbal area with mixture of whitish and dark-brown scales; wing between crescent line and limbal zone whitish in Sc + R1–M3, and with beige scales and bold rusty-brown lunules in spaces CuA1–2A.

Male genitalia (Figure 3A). Falx stout; lobes of uncus rather long, with well-developed inner processes; genital capsula elongated; vinculum broadened, with well-developed inner lobes; valva covered with hairs of different lengths, robust, with very broad ovoid basal part reaching vinculum and abruptly turning into distal part as long as 1.5 lengths of basal part; proximal half of distal part of valva with nearly parallel sides; distal half of valva in outer edge tapering to rounded apex; in bilobed configuration of valvae, their distal parts nearly gradually tapering after medial flexure; valvae strongly jointed, with small groove between apices; saccus triangular with rounded apex, 1/4 as long as genitalia; aedeagus rather slender, arcuate, about 1.8 times as long as genital capsule, with serrated slightly deflected cornuti.

Female (Figure 2B). Generally similar to male but blue field broader, ventral side lighter and less contrasted. Forewing length 16.0 mm.

Female genitalia (Figure 4A). Lamella postvaginalis very long and broad, semicircular; anterior edge with small depression; lamella antevaginalis small, membranous; junctures of lamellae well-developed; ductus bursae with slightly broadened antrum, two times as long as lamella postvaginalis, slightly contracting towards bursa; corpus bursae with two very small two-spined signi.

Etymology. The new species is named after the Hmong people, the largest ethnic group of Ha Giang Province. The type locality of the new species is situated near Sa Phin commune, which was the site of the residence of the Hmong kings.

Distribution and biology. *C.* (*A.*) *hmong* sp. n. is known only from the type locality in Dong Van Karst Plateau in Northeastern Vietnam, Ha Giang Province, about 450 km south from the type locality of the somewhat morphologically similar species *C.* (*A.*) *bijieensis*. The new species inhabits the middle mountain zone at 1400–1600 m a.s.l.

#### 3.2.2. *Callophrys* (*Ahlbergia*) *tay* Krupitsky, Shapoval & Shapoval, sp. n. (Figure 2C–E, Figure 3B and Figure 4B)

LSID urn:lsid:zoobank.org:act:9B50FE30-1F24-4EDC-A42D-DC73B5F9096B.

Type material. Holotype: ♂, specimen code CAL122, GenBank accession number OM630557, North Vietnam, Ha Giang Province, Dong Van District, Dong Van Karst Plateau, Sa Phin commune, 1400–1600 m a.s.l., 23°15′11″ N 105°15′01″ E, April 2021, local collector leg. (ZMMU). Paratypes: 6 ♂ (CAL124, OM630554; CAL131, OM630555; CAL136, OM630551; CAL144; CAL145; CAL146), same data as holotype (CEDM); ♂, North Vietnam, Ha Giang Province, Dong Van District, Dong Van Karst Plateau, April 2003, local collector leg. (CVTM); 2 ♀ (CAL121, OM630552; CAL138, OM630556), same data as holotype (CEDM).

##### Diagnosis

According to our phylogenetic analysis, the species genetically closest to *C.* (*A.*) *tay* sp. n. is *C.* (*A.*) *clarolinea*; the genetic divergence is 0.80% ± 0.33%. The only species somewhat similar to this new species is *C.* (*Novosatsuma*) *monstrabila* (Johnson, 1992), comb. nov., the type species of the genus *Novosatsuma* sensu Johnson (1992), known with certainty from the sole holotype. The new species differs from the latter in narrower deep-blue field limited by postdiscal area on dorsal side of forewing (vs. larger light silvery-blue field reaching submarginal area in *C.* (*N.*) *monstrabila*, cf. Figure 85 in [1]), uniformly grey or light-grey basal and postdiscal bands (vs. totally darkened basal band and darkened postdiscal band in spaces M3–CuA2, with only light spots in spaces Sc + R1 and 2A, in *C.* (*N.*) *monstrabila*, cf. Figure 85 in [1]), valva with slightly broadened basal part and distal parts nearly gradually tapering to apices in bilobed configuration (vs. strongly broadened basal part of valva abruptly turning into distal part with sides situated parallel along most of valva length and tapering near apices in bilobed configuration in *C.* (*N.*) *monstrabila*).

##### Description

Male (Figure 2C,E). Head: antenna black, white-ringed at bases of segments, antennal club black; eye surrounded with white stripe, dark brown with very short sparse hairs; head dark brown, with white scales and hairs; palpus: palpomere 2 black with whitish hairs and scales on outside, white inside, palpomere 3 black.

Thorax: dark brown with blueish-grey hairs dorsally, densely covered with grey hairs ventrally; femur dark brown, tibia and tarsus black with white stripes dorsally, whitish ventrally.

Abdomen: dark brown with grey scales and hairs dorsally, whitish ventrally.

Forewing: triangular, with pointed apex and rounded termen. Forewing length 12.0 mm in holotype, 12.0–14.0 mm in paratypes. Dorsal side dark brown, cell and discal area in spaces M3–2A densely covered with dark-blue scales; veins dark brown; outer margin coloured as background; fringe wavy, convex at veins, dark brown with admixture of whitish scales; basal area of wing and inner margin covered with rather long whitish hairs, tornus with long dark-brown scales; androconial spot narrow, lanceolate, brown to dark brown, length about 2.0–2.5 mm. Ventral side brown to greyish brown, densely covered with grey scales, base and costa dark brown; postmedial line wavy, dark brown with grey scales distally; crescent line more or less developed in spaces R3–CuA1, consisting of dark-brown elements; submarginal area with grey scales; outer margin dark brown; fringe as on dorsal side.

Hindwing: costa nearly straight, apex rounded, termen wavy, anal lobe well-developed, rounded. Dorsal side dark brown with broad dark-blue field in spaces Rs–CuA2 extending to postdiscal or submarginal area in spaces M3–CuA2; veins black; spaces 2A–3A; outer margin black, bordered by blue scales, more intensively in spaces M3–CuA2, and with grey scales in half of space CuA2 and 2A near anal lobe; basal half of wing and inner margin covered with long, sparse, whitish hairs; fringe dark brown proximally, whitish distally, with long black scales at veins; anal lobe with long black scales. Ventral side with dark-brown base and grey spots near postbasal marks, postbasal marks and marginal band of disc bold, black, discal band strongly darkened, especially in spaces CuA2–3A; postdiscal band grey, varying from light grey to grey-brown; crescent line black with grey scales, usually well-developed in spaces M3–2A, traced in other spaces; limbal area with mixture of whitish and dark-brown scales; wing between crescent line and limbal zone as postdiscal band in Sc + R1–M3, and with darker scales and bold brown lunules in spaces CuA1–2A, anal lobe with large brown spot; fringe as on dorsal side.

Male genitalia (Figure 3B). Falx rather narrow, pointed; lobes of uncus rather short; genital capsule rather compact; vinculum broadened, with well-developed inner lobes reaching distal part of valva; valva covered with hairs of different length, narrow, with slightly broadened basal part barely reaching vinculum and distal part 1.5 times as long as basal part; in bilobed configuration of valvae, their distal parts nearly gradually tapering after small medial flexure, apices of valvae disjoined; saccus triangular with rounded apex, as long as 1/5 of genitalia length; aedeagus rather slender, slightly curved mesially, about 1.5 times as long as length of genital capsule, with small serrated slightly deflected cornuti.

Female (Figure 2D). Generally similar to male but blue field broader and ventral colouration lighter, ground colour light grey, discal band of disc and limbal area grey. Forewing length 14.0–15.0 mm.

Female genitalia (Figure 4B). Lamella postvaginalis semicircular, strongly deflected dorsally; lamella antevaginalis large, membranous; junctures of lamellae well-developed, very large; ductus bursae with broadened antrum, about two times as long as lamella postvaginalis, straight; corpus bursae with two large two-spined signi with spines of equal length.

Etymology. The new species is named after the Tay people, the second-largest ethnic group of Ha Giang Province.

Distribution. The new species is known only from the type locality in Dong Van Karst Plateau in Northeastern Vietnam, Ha Giang Province, about 1100 km southeast from the somewhat morphologically similar species *C.* (*N.*) *monstrabila* described from the Naga Hills on the border of India and Myanmar. *C.* (*A.*) *tay* sp. n. inhabits the middle mountain zone at 1400–1600 m a.s.l.

#### 3.2.3. *Callophrys* (*Cissatsuma*) *devyatkini* Krupitsky, Shapoval & Shapoval, sp. n. (Figure 2F,G, Figure 3C and Figure 4C)

LSID urn:lsid:zoobank.org:act:AD6F3F89-8951-4BA0-A471-83FC0EABCFB7.

Type material. Holotype: ♀, specimen code CAL069, GenBank accession number OM630566, North Vietnam, Ha Giang Province, Dong Van District, Dong Van Karst Plateau, Sa Phin commune, 1400–1600 m a.s.l., 23°15′11″ N 105°15′01″ E, May 2018, local collector leg. (ZMMU). Paratypes: ♂ (CAL070, OM630567), same data as holotype, local collector leg. (CEDM); 3 ♂ (CAL143, OM630568; CAL127, OM630569; CAL149), North Vietnam, Ha Giang Province, Dong Van District, Dong Van Karst Plateau, Sa Phin commune, 1400–1600 m a.s.l., 23°15′11″ N 105°15′01″ E, April 2021, local collector leg. (CEDM).

##### Diagnosis

According to our phylogenetic analysis, the species genetically closest to *Callophrys* (*Cissatsuma*) *devyatkini* sp. n. is *C.* (*Cissatsuma*) *berezowskii* (Krupitsky, 2018), comb. nov.; genetic divergence is 0.53 ± 0.26%. The new species clearly differs from the known species of elfin butterflies, except for the somewhat similar *C.* (*Cissatsuma*) *zhoujingshuae* (Huang & Zhou, 2014), comb. nov., described from Shaanxi Province, China, though *C.* (*C.*) *devyatkini* sp. n. can be easily distinguished from the latter by the developed androconia (androconia absent in *C.* (*C.*) *zhoujingshuae*), dark-brown dorsal side of the wings with deep-blue field (vs. nearly black dorsal side of the wings with celestial-blue field in *C.* (*C.*) *zhoujingshuae*), brown ventral side of the forewing with wavy postmedial line (vs. ochre ventral side of the forewing with U-shaped postmedial line in *C.* (*C.*) *zhoujingshuae*), less contrasted ventral side of the hindwing (vs. strongly contrasted ventral side of the hindwing with dark-brown disc and crescent line in *C.* (*C.*) *zhoujingshuae*) and deep indentation of the margin of the disc in spaces M1–M2 (vs. smoother margin of the disc in the spaces M1–M2 in *C.* (*C.*) *zhoujingshuae*). In the male genitalia, the new species differs from *C.* (*C.*) *zhoujingshuae* in the valva with the basal part as long as the distal part and the distal part with lateral projection (the basal part 1/3 as long as the valva, the lateral side of the valva straight in *C.* (*C.*) *zhoujingshuae*). In the female genitalia, the new species differs from *C.* (*C.*) *zhoujingshuae* in the lamella postvaginalis with the anterior edge bearing a large semicircular depression and very well-developed large junctures of lamellae (vs. the lamella postvaginalis lacking a depression on the anterior edge, the junctures of the lamellae smaller in *C.* (*C.*) *zhoujingshuae*) and very small signum with poorly developed spine (vs. large signum with well-developed long spine in *C.* (*C.*) *zhoujingshuae*).

Additionally, the male of *C.* (*C.*) *devyatkini* sp. n. somewhat resembles *C*. (*Ahlbergia*) *chalcidis* comb. nov., described from Kunming, Yunnan Province, China. The new species differs from the latter in terms of the less contrasted ventral surface of both wings, the shape of the margin of the disc of the hindwing as well as the valva with larger broadened basal part and slightly curved distal part (smaller, slightly broadened basal part and curved distal part of valva in *C*. (*A.*) *chalcidis*). The female of the new species is quite different both in colouration and the genitalia structure compared to the female of *C*. (*A.*) *chalcidis* (e.g., Figures 67, 68, 102 and 111 in [13]) as well as to other known Palaearctic elfin butterflies.

##### Description

Female (Figure 2G). Head: antenna brown, white-ringed at bases of segments, antennal club brown; eye surrounded with white stripe, light brown with very short sparse hairs; frons with brown hairs, top of head with brown scales and hairs; palpus brown with admixture of whitish hairs and scales.

Thorax: brown with whitish hairs dorsally, densely covered with rusty-brown and whitish hairs ventrally; legs brown with light-brown scales.

Abdomen: brown with whitish hairs dorsally, whitish ventrally, tip rusty brown.

Forewing: triangular, with pointed apex and rounded convexity at cells M3–CuA1. Forewing length 15.0 mm. Dorsal side dark brown; basal, postbasal and discal area except costal zone covered with blue scales; outer margin same as background; fringe dark brown, with admixture of rusty-brown scales, wavy, with projections at veins; basal area of wing and inner margin covered with rather long whitish hairs. Ventral side motley; cell and spaces R1–CuA1 rusty brown before postmedial line, brown in other spaces; postmedial line wavy, rusty brown with whitish scales distally; marginal area with traces of dark-brown crescent line surrounded with whitish scales; wing covered with whitish and light-brown scales, more densely near costa and after postmedial line; fringe as on dorsal side.

Hindwing: costa straight, apex and termen rounded. Dorsal side dark brown with large steel-blue field and diffused blue scales mostly limited by postdiscal area and penetrated by dark-brown veins with large blue field mostly limited by postdiscal area and penetrated by dark-brown veins, spaces 2A–3A light brown with admixture of greyish-blue scales, outer margin dark brown bordered by blue scales, most intensively in spaces CuA1–2A; basal half of wing and inner margin covered with long sparse whitish hairs; apical and anal lobes well-developed, prominent, rusty brown; fringe wavy, dark-brown and rusty proximally, whitish distally, dark at convexities near veins.

Ventral side: base rusty brown; postbasal marks well-developed, wavy, dark brown, with beige scales proximally; marginal band of disc strongly uneven, rusty brown, consisting of blurred spot in spaces Sc + R1 and Rs, two parallel straight brown lines in spaces M1–M2, projection in space M3 and bold wavy line in spaces CuA1–2A; space between postbasal and marginal band of disc brown with beige scales; postdiscal area motley, with beige, whitish, brown and rusty-brown scales; crescent line uneven, consisting of groups of brown and rusty-brown scales; submarginal (limbal) area dark brown with whitish scales, separated from crescent line by beige and rusty-brown scales; anal lobe with rusty-brown spot; outer margin dark brown; fringe as on dorsal side; most of wing covered with long whitish and greyish hairs.

Female genitalia (Figure 4C). Lamella postvaginalis long and broad, trapezoid with rounded corners and two lateral convolutions; anterior edge with large semicircular depression; lamella antevaginalis small, membranous; junctures of lamellae well-developed; ductus bursae rather stout and long, three times as long as lamella postvaginalis, with slightly broadened antrum and base; corpus bursae with two small unispined signi.

Male (Figure 2F). Forewing length 12.0–14.0 mm. Generally similar to female, but blue field narrower, especially on forewing, cell blue-grey with admixture of blue scales, discal area in spaces CuA2–2A with blue scales; veins dark brown; costal area with suffusion of beige scales, more intensive near base. Androconial spot very thin, lanceolate, brown, length about 2.0 mm. Hindwing with large steel-blue field and diffused blue scales mostly limited by postdiscal area and penetrated by dark-brown veins. In one specimen, spaces M1–M2 with one straight brown line.

Male genitalia (Figure 3C). Falx stout; lobes of uncus broad, rather short; genital capsula rather large; valva covered with hairs of different lengths, robust, with ovoid basal part abruptly turning into distal part 1.5 times as long as basal part; in bilobed configuration of valvae, their distal parts nearly gradually tapering after medial flexure, apices of valvae broadly disjointed; saccus triangular with thin oblong apex, 1/4 times as long as genitalia length; aedeagus rather stout, arcuate, about two times as long as genital capsule, with serrated cornuti; lower cornutus strongly deflected.

Etymology. The new species is named in memory of Alexey Devyatkin (1957–2012), Russian lepidopterist, renowned specialist in the Oriental Hesperiidae and the Rhopalocera of Vietnam.

Distribution and biology. *Callophrys* (*Cissatsuma*) *devyatkini* sp. n. is known only from the type locality in Dong Van Karst Plateau in Northeastern Vietnam, Ha Giang Province, nearly 1500 km south from the most morphologically similar species, *C.* (*C.*) *zhoujingshuae.* The new species inhabits the middle mountain zone at 1400–1600 m a.s.l.

#### 3.2.4. *Callophrys* (*Ahlbergia*) *dao* Krupitsky, Shapoval & Shapoval, sp. n. (Figure 2H and Figure 4D)

LSID urn:lsid:zoobank.org:act:63120BE3-C61D-4B69-B618-8A72239DBB0C.

Type material. Holotype: ♀, specimen code CAL137, GenBank accession number OM641843, North Vietnam, Ha Giang Province, Dong Van District, Dong Van Karst Plateau, Sa Phin commune, 1400–1600 m a.s.l., 23°15′11″ N 105°15′01″ E, April 2021, local collector leg. (ZMMU).

##### Diagnosis

Despite the *COI* barcode shared with *C.* (*A.*) *tay* sp. n., such characters as the rounded hindwing nearly entirely covered with blue scales and inwardly directed rounded anal lobe of hindwing place *C.* (*A.*) *dao* sp. n. into the *C.* (*A.*) *leechi* (de Nicévile, 1893), comb. nov. species group comprising three species, namely, *C.* (*A.*) *leechi* from Northeast India, *C.* (*A.*) *nicevillei* (Leech, 1893), comb. nov. from East China and *C.* (*A.*) *liyufeii* (Huang & Zhou, 2014) comb. nov. known from Shaanxi Province, China. The new species differs from the former two species in the well-developed contrasted pattern of the dorsal side of the hindwing (uncontrasted hindwing with blurred patter in *C.* (*A.*) *leechi* and *C.* (*A.*) *nicevillei*). Externally, *C.* (*A.*) *dao* sp. n. differs from *C.* (*A.*) *liyufeii* in strongly contrasted ventral side of the hindwing with dark-brown margin of disc, crescent line and limbal area (less contrasted ventral side of the hindwing with poorly developed light-brown crescent line and limbal area in *C.* (*A.*) *liyufeii*), V-shaped incision of the margin of the disc at M2 (the margin of the disc straight at M2 in *C.* (*A.*) *liyufeii*). In the female genitalia, the new species differs from *C.* (*A.*) *liyufeii* in broad trapezoid lamella postvaginalis and short ductus bursae (narrower rounded lamella postvaginalis with longer ductus bursae in *C.* (*A.*) *liyufeii*).

##### Description

Female (Figure 2H). Head: antenna broken off; eye surrounded with white stripe, dark brown with very short sparse hairs; head dark brown, with white scales and hairs; palpus: palpomere 2 black with white hairs and scales, palpomere 3 black.

Thorax: brown with blueish-grey hairs dorsally, densely covered with grey hairs ventrally; leg rusty brown with whitish scales.

Abdomen: brown with grey scales and hairs dorsally, whitish ventrally.

Forewing: triangular, with rounded termen and apex; forewing length 14.0 mm. Dorsal side dark brown, cell and discal area in spaces M3–2A densely covered with light-blue scales; veins dark brown; outer margin same as background; fringe slightly wavy, convex at veins, dark brown with admixture of whitish scales; basal area of wing and inner margin covered with rather long whitish hairs, tornus with long dark-brown scales. Ventral side rusty brown, darker at base, with whitish scales along inner margin; cell marking brown; postmedial line wavy, rusty brown with scarce whitish scales distally; crescent line absent; submarginal area with dark-brown scales and whitish scales in CuA2–2A; outer margin rusty brown; fringe as on dorsal side.

Hindwing: costal area with small basal lobe, nearly straight, apex rounded, termen wavy, anal lobe well-developed, rounded. Dorsal side grey-brown with very broad light-blue field covering nearly entire wing surface except postdiscal area in cells Sc + R1 and submarginal area in cells Rs–M1 covered with grey-brown scales, cells 2A–3A covered with blueish-grey scales; veins grey-brown; outer margin black, bordered by blue scales, more intensively in spaces M3–CuA2, and with grey-blue scales in half of space CuA2 and 2A near anal lobe; basal half of wing and inner margin covered with long sparse whitish hairs; fringe dark-brown proximally, whitish distally, with long black scales at veins; anal lobe with long black scales. Ventral side with dark rusty-brown base, rusty brown near postbasal marks, postbasal marks blurred, brown, discal band darkened, with whitish scales in space Sc + R1, with rusty-brown, brown and dark-brown scales in other spaces; marginal band of disc strongly uneven, consisting of blurred dark-brown spots in spaces Sc + R1–M2, strong projection in space M3 and bold wavy dark-brown line in spaces CuA1–2A; postdiscal band rusty brown with whitish scales in spaces 2A–3A; crescent line blurred, dark brown, well-developed in spaces M3–2A, traced in other spaces; limbal area with dark-brown scales and admixture of whitish scales; wing between crescent line and limbal area rusty brown; anal lobe with rusty-brown spot; outer margin black; fringe as on dorsal side; basal half of wing and inner margin covered with short light hairs.

Female genitalia (Figure 4D). Lamella postvaginalis broad, trapezoid with rounded corners; lamella antevaginalis small, membranous; junctures of lamellae small; ductus bursae with slightly broadened antrum, about two times as long as lamella postvaginalis, straight, gradually broadened to base; corpus bursae with two very small two-spined signi.

Etymology. The new species is named after the Dao people, the third-largest ethnic group of Ha Giang Province.

Distribution. The new species is known only from the type locality in Dong Van Karst Plateau in Northeastern Vietnam, Ha Giang Province, about 1500 km south from the somewhat morphologically similar *C.* (*A.*) *liyufeii* described from Baoji City, Shaanxi Province, China.

## 4. Discussion

Northeastern Vietnam (including Ha Giang Province, where the type locality of all four species described in this paper is situated) east of the Red River, a major biogeographical boundary, is known as a biodiversity hotspot. It hosts a number of endemic species of butterflies, including Lycaenidae, or species endemic to a region common for the mountains of Northeastern Vietnam and Guangxi Province in China [28,42]. The discovery of four new species of the Palaearctic elfin butterflies in Vietnam is important in terms of biogeography: it expands the range of the group towards the southeast, beyond the Palaearctic region, and clarifies some aspects of its distribution (Figure 5).

According to the morphological characters mentioned in the diagnosis and our phylogenetic analysis, *C.* (*A.*) *hmong* sp. n. is closely related to the Chinese species *C.* (*A.*) *clarolinea* and *C.* (*A.*) *bijieensis.* It is geographically isolated from both species by the rather low eastern part of the Yunnan–Guizhou Plateau, which is characterised by a mostly subtropical climate unsuitable for such temperate insects as elfin butterflies. *C.* (*A.*) *hmong* sp. n. or a closely related new species also can be found in the mountains of Laos and Northern Thailand, e.g., the elfin butterfly specimen from Chiang Mai mentioned by Ek-Amnuay [24] as “*Callophrys* sp.” shares some diagnostic characters of the species in question and probably belongs to an undescribed species.

*Callophrys* (*Ahlbergia*) *tay* sp. n. shares some details of the colouration with *C.* (*N.*) *monstrabila*, the type species of the genus *Novosatsuma* sensu Johnson [1], known with certainty from the sole holotype from the Naga Hills on the border of India and Myanmar. Such an affinity is peculiar in terms of biogeography, as the Naga Hills and the type locality of the latter new species in Ha Giang Province are separated by a distance of over 1000 km. In its turn, the darkened basal disc, the light spot in cell 2A and the shape of the valva in the male genitalia make *C.* (*N.*) *monstrabila* close to *C*. (*A.*) *chalcidis* described from Yunnan Province, China. In this case, *C*. (*A.*) *tay* sp. n. and *C*. (*A.*) *chalcidis* are members of the subgenus *Novosatsuma*. If these species are actually closely related, *Novosatsuma* sensu Johnson [1] is a polyphyletic group, as *C*. (*A.*) *tay* sp. n. and *C*. (*N.*) *collosa*, *C*. (*N.*) *prodiga* and *C*. (*N.*) *magnapurpurea* are placed in different clades in our phylogenetic analysis and must be treated under different subgenera. As noted by Huang and Zhou [11], the difference in the male genitalia between *Ahlbergia* and *Novosatsuma* is not clearly marked, and the differences in the female genitalia are overvalued. A robust ductus bursae with a well-defined fluted antrum is the most important diagnostic character of the genus *Novosatsuma* sensu Johnson [1], but this character was not based on the type species of the genus (as it was known only from the male holotype). The female genitalia of *C*. (*A.*) *tay* sp. n. do not possess any diagnostic characters at the generic or subgeneric level and do not fit in with Johnson’s concept of *Novosatsuma*, so the status of this taxon remains questionable and requires further study with more species involved in the phylogenetic analysis.

Unlike the latter case, the results of our study do not reject the concept of *Cissatsuma* sensu Johnson [1], but this clade appears polyphyletic, as the morphologically different *C*. (*A.*) *frivaldszkyi* is united with the *Cissatsuma* species in our analysis. The type species of *Cissatsuma*, *C.* (*Cissatsuma*) *albilinea* (Riley, 1939), comb. nov., is rare in collections and was inaccessible for the phylogenetic analysis, but we analysed the recently described morphologically close species *C*. (*C.*) *berezowskii*.

In the case of *C.* (*C.*) *devyatkini* sp. n., its placement in a clade together with *C*. (*C.*) *berezowskii* is supported by the morphologies of the male and female genitalia. The genitalia of both species, as well as those of *C*. (*C.*) *zhoujingshuae*, which are somewhat similar to those of *C.* (*C.*) *devyatkini* sp. n., fit the diagnosis of *Cissatsuma* by Johnson [1]: the valvae in ventral view with straight or concaved lateral margins in most parts, except at the apex, with the ductus bursae robust and relatively wider and longer than in *Ahlbergia*. As in the case of *C.* (*A.*) *hmong* sp. n., the new *Cissatsuma* species is isolated from *C*. (*C.*) *zhoujingshuae* by the eastern part of the Yunnan–Guizhou Plateau.

According to the morphological characters, *C.* (*A.*) *dao* sp. n. belongs to the *C.* (*A.*) *leechi* species group. Based on the pattern of the dorsal side of the hindwing, it is related to *C.* (*A.*) *liyufeii* from Shaanxi Province, China, from which it is geographically isolated by the rather low eastern part of the Yunnan–Guizhou Plateau.

Although the *COI*-based phylogenetic analysis does not resolve relations of all involved groups, it reveals that the genus *Callophrys* sensu stricto is also polyphyletic. The polyphyly of the Palaearctic *Callophrys* sensu stricto was demonstrated earlier by ten Hagen and Miller [19]. In their neighbour-joining tree, *C*. (*Callophrys*) *avis* was a sister species to *C*. (*Cissatsuma*) *tuba*, and *C*. (*A.*) *frivaldszkyi* was included in the *C*. (*Callophrys*) *rubi* clade. Surprisingly, in our reconstruction, *C*. (*C*.) *avis* is united with the type species of *Ahlbergia*, *C*. (*A.*) *ferrea*. This result can be explained by the connected range of the ancestor of the group in the past. The same example is known in the tribe Theclini of the subfamily Theclinae: the monotypic Mediterranean genus *Laesopis* Rambur, 1858 is morphologically similar to the East Asian genus *Artopoetes* Chapman, 1909 [43]. Despite the generally similar colouration and wing pattern, the polyphyly of the genus *Callophrys* sensu stricto is also supported by the morphologies of the genitalia of *C*. (*C*.) *avis*, *C*. (*C*.) *mystaphia* and *C*. (*C*.) *mystaphioides*, which are characterised by the complicated valva structure in the male genitalia and the very simple lamella postvaginalis appearing similar to those of the Palaearctic elfin butterflies and some Nearctic *Callophrys* [44,45]. As the recent genome-wide phylogenetic analysis of the Nearctic *Callophrys* has shown, a similar wing pattern can be implemented in unrelated clades of this group independently [18], such as the green colouration of the Palaearctic *Callophrys* sensu lato and species of the recently established Nearctic subgenus *Greenie* Grishin, 2021.

Values of the interspecific uncorrected pairwise genetic divergences (*p*-distances) of the studied *Callophrys* (sensu lato) species ranging from 0.15% to 2.72% are less than the species threshold of about 3% empirically found for Lepidoptera in general [46]. In the subfamily Theclinae, shared or very close *COI* mitochondrial barcodes in morphologically different species were also found in the Palaearctic *Callophrys* sensu lato [19], as well as in the *Tomares* hairstreaks [47]. This pattern can be explained by incomplete lineage sorting or hybridisation leading to mitochondrial introgression, which occurs occasionally in Lycaenidae [48,49,50], including the genus *Callophrys* sensu lato [22]. The mitochondrial introgression from *C.* (*A.*) *tay* sp. n. to *C.* (*A.*) *dao* sp. n. belonging to the morphologically distinct *C.* (*A.*) *leechi* species group is an explanation of the shared *COI* mitochondrial barcode.

Another possible explanation of the shared or very close mitochondrial haplotypes within the studied group is an influence of the rickettsial bacterium *Wolbachia* Hertig, 1936, an endosymbiont associated with mitochondria and thus maternally inherited. *Wolbachia* can cause selective sweeps in mitochondrial haplotypes owing to genetic hitchhiking, leading to mitochondrial introgression and reduced mitochondrial diversity [51].

## 5. Conclusions

Our results demonstrate a need for a complex analysis of genetic markers and morphology for the delimitation and taxonomic rearrangement of the Palaearctic elfin butterflies and *Callophrys*. The final taxonomic solution of the subgeneric classification should be based on a multilocus molecular phylogenetic analysis covering all the species groups of the Palaearctic *Callophrys* sensu lato and provide a robust phylogenetic scheme for this genus.

## Figures and Tables

**Table 1:** Specimens of the genus *Callophrys* studied in the current research and their *COI* GenBank accession numbers. Specimens with genitalia dissected are marked with asterisks. HT, holotype; PT, paratype; n/a, not available. GenBank accession numbers of the specimens obtained in the current study are in bold.

Code	Genus	Subgenus	Species	Locality	Accession No.	Note
CAL141 *	*Callophrys*	*Ahlbergia*	*hmong* sp. n.	VIETNAM, Ha Giang Prov., Dong Van	**OM630550**	HT
CAL071 *	*Callophrys*	*Ahlbergia*	*hmong* sp. n.	VIETNAM, Ha Giang Prov., Dong Van	**OM630537**	PT
CAL123	*Callophrys*	*Ahlbergia*	*hmong* sp. n.	VIETNAM, Ha Giang Prov., Dong Van	**OM630538**	PT
CAL125 *	*Callophrys*	*Ahlbergia*	*hmong* sp. n.	VIETNAM, Ha Giang Prov., Dong Van	**OM630539**	PT
CAL126 *	*Callophrys*	*Ahlbergia*	*hmong* sp. n.	VIETNAM, Ha Giang Prov., Dong Van	**OM630540**	PT
CAL128 *	*Callophrys*	*Ahlbergia*	*hmong* sp. n.	VIETNAM, Ha Giang Prov., Dong Van	**OM630541**	PT
CAL129	*Callophrys*	*Ahlbergia*	*hmong* sp. n.	VIETNAM, Ha Giang Prov., Dong Van	**OM630542**	PT
CAL130	*Callophrys*	*Ahlbergia*	*hmong* sp. n.	VIETNAM, Ha Giang Prov., Dong Van	**OM630543**	PT
CAL132	*Callophrys*	*Ahlbergia*	*hmong* sp. n.	VIETNAM, Ha Giang Prov., Dong Van	**OM630544**	PT
CAL133	*Callophrys*	*Ahlbergia*	*hmong* sp. n.	VIETNAM, Ha Giang Prov., Dong Van	**OM630545**	PT
CAL134	*Callophrys*	*Ahlbergia*	*hmong* sp. n.	VIETNAM, Ha Giang Prov., Dong Van	**OM630546**	PT
CAL135 *	*Callophrys*	*Ahlbergia*	*hmong* sp. n.	VIETNAM, Ha Giang Prov., Dong Van	**OM630547**	PT
CAL139	*Callophrys*	*Ahlbergia*	*hmong* sp. n.	VIETNAM, Ha Giang Prov., Dong Van	**OM630548**	PT
CAL140	*Callophrys*	*Ahlbergia*	*hmong* sp. n.	VIETNAM, Ha Giang Prov., Dong Van	**OM630549**	PT
CAL147	*Callophrys*	*Ahlbergia*	*hmong* sp. n.	VIETNAM, Ha Giang Prov., Dong Van	n/a	PT
CAL148	*Callophrys*	*Ahlbergia*	*hmong* sp. n.	VIETNAM, Ha Giang Prov., Dong Van	n/a	PT
CAL122 *	*Callophrys*	*Ahlbergia*	*tay* sp. n.	VIETNAM, Ha Giang Prov., Dong Van	**OM630557**	HT
CAL096 *	*Callophrys*	*Ahlbergia*	*tay* sp. n.	VIETNAM, Ha Giang Prov., Dong Van	**OM630553**	PT
CAL121 *	*Callophrys*	*Ahlbergia*	*tay* sp. n.	VIETNAM, Ha Giang Prov., Dong Van	**OM630552**	PT
CAL124 *	*Callophrys*	*Ahlbergia*	*tay* sp. n.	VIETNAM, Ha Giang Prov., Dong Van	**OM630554**	PT
CAL131 *	*Callophrys*	*Ahlbergia*	*tay* sp. n.	VIETNAM, Ha Giang Prov., Dong Van	**OM630555**	PT
CAL136 *	*Callophrys*	*Ahlbergia*	*tay* sp. n.	VIETNAM, Ha Giang Prov., Dong Van	**OM630551**	PT
CAL138 *	*Callophrys*	*Ahlbergia*	*tay* sp. n.	VIETNAM, Ha Giang Prov., Dong Van	**OM630556**	PT
CAL142	*Callophrys*	*Ahlbergia*	*tay* sp. n.	VIETNAM, Ha Giang Prov., Dong Van	n/a	PT
CAL144	*Callophrys*	*Ahlbergia*	*tay* sp. n.	VIETNAM, Ha Giang Prov., Dong Van	n/a	PT
CAL145	*Callophrys*	*Ahlbergia*	*tay* sp. n.	VIETNAM, Ha Giang Prov., Dong Van	n/a	PT
CAL146	*Callophrys*	*Ahlbergia*	*tay* sp. n.	VIETNAM, Ha Giang Prov., Dong Van	n/a	PT
CAL137 *	*Callophrys*	*Ahlbergia*	*dao* sp. n.	VIETNAM, Ha Giang Prov., Dong Van	**OM641843**	HT
GS119 *	*Callophrys*	*Ahlbergia*	*clarolinea* comb. nov.	CHINA, NW Yunnan, Lijiang	**OM630558**	
GS120 *	*Callophrys*	*Ahlbergia*	*clarolinea* comb. nov.	CHINA, NW Yunnan, Lijiang	**OM630559**	
CAL065	*Callophrys*	*Ahlbergia*	*ferrea*	JAPAN, Hokkaido, Kato-gun	**OM630560**	
CAL066	*Callophrys*	*Ahlbergia*	*ferrea*	JAPAN, Hokkaido, Kato-gun	**OM630561**	
CAL069 *	*Callophrys*	*Cissatsuma*	*devyatkini* sp. n.	VIETNAM, Ha Giang Prov., Dong Van	**OM630566**	HT
CAL070 *	*Callophrys*	*Cissatsuma*	*devyatkini* sp. n.	VIETNAM, Ha Giang Prov., Dong Van	**OM630567**	PT
CAL143 *	*Callophrys*	*Cissatsuma*	*devyatkini* sp. n.	VIETNAM, Ha Giang Prov., Dong Van	**OM630568**	PT
CAL127	*Callophrys*	*Cissatsuma*	*devyatkini* sp. n.	VIETNAM, Ha Giang Prov., Dong Van	**OM630569**	PT
CAL149 *	*Callophrys*	*Cissatsuma*	*devyatkini* sp. n.	VIETNAM, Ha Giang Prov., Dong Van	n/a	PT
CAL072 *	*Callophrys*	*Cissatsuma*	*berezowskii* comb nov.	CHINA, Sichuan Prov., Aba Pref., Chuanzhusi	**OM630564**	
CAL073 *	*Callophrys*	*Cissatsuma*	*berezowskii* comb nov.	CHINA, Sichuan Prov., Aba Pref., Chuanzhusi	**OM630565**	
CAL074 *	*Callophrys*	*Ahlbergia*	sp.	CHINA, Sichuan Prov., Wenchuan env.	**OM630562**	
CAL075 *	*Callophrys*	*Ahlbergia*	sp.	CHINA, Sichuan Prov., Jinchuan County	**OM630563**	
CAL094 *	*Callophrys*	*Novosatsuma*	*prodiga* comb. nov.	CHINA, Yunnan Prov., Dali	**OM630570**	
CAL095 *	*Callophrys*	*Novosatsuma*	*collosa* comb. nov.	CHINA, Shaanxi Prov., Taibaishan Mts.	**OM630572**	
CAL097 *	*Callophrys*	*Novosatsuma*	*magnapurpurea* comb. nov.	CHINA, Yunnan Prov., Wumeng Mt.	**OM630571**	
	*Callophrys*	*Ahlbergia*	*frivaldszkyi*	RUSSIA, Omsk Oblast	OL457026	
	*Callophrys*	*Ahlbergia*	*frivaldszkyi*	RUSSIA, Irkutsk Oblast	MW785858	
	*Callophrys*	*Ahlbergia*	*frivaldszkyi*	RUSSIA, Irkutsk Oblast	MW785859	
	*Callophrys*	*Callophrys*	*rubi*	MOROCCO, Ifrane	MT260503	
	*Callophrys*	*Callophrys*	*rubi*	RUSSIA, Moscow Oblast	MW502280	
	*Callophrys*	*Callophrys*	*avis*	SPAIN, Barcelona	GU676347	
	*Callophrys*	*Callophrys*	*avis*	SPAIN, Barcelona	GU676532	
	*Callophrys*	*Cisincisalia*	*johnsoni*	USA	JN000833	
	*Callophrys*	*Incisalia*	*augustinus*	USA, California	HQ561190	
	*Callophrys*	*Incisalia*	*henrici*	USA, Maryland	KP150273	
	*Callophrys*	*Incisalia*	*eryphon*	USA, Colorado	HQ583526	
	*Callophrys*	*Incisalia*	*niphon*	USA, Maryland	KP150297	
	*Callophrys*	*Incisalia*	*irus*	USA, Maryland	KP150284	
	*Callophrys*	*Incisalia*	*polios*	CANADA, Manitoba	KT129894	
	*Callophrys*	*Mitoura*	*gryneus*	CANADA, Ontario	KT133344	
	*Neolycaena*	*Rhymnaria*	*baidula*	KYRGYZSTAN, Inner Tian Shan	MW785936	

**Table 2:** Primers used in this study.

Primer Pair	Fragment Length (bp)	Reference
LepF (ATTCAACCAATCATAAAGATATTGG) LepR (TAAACTTCTGGATGTCCAAAAAATCA)	658	[33]
LCO1490 (GGTCAACAAATCATAAAGATATTGG) HCO2198 (TAAACTTCAGGGTGACCAAAAAATCA)	658	[32]
LepF (ATTCAACCAATCATAAAGATATTGG) MH-MR1 (CCTGTTCCAGCTCCATTTTC)	307	[34]
LCO1490 (GGTCAACAAATCATAAAGATATTGG) MH-MR1 (CCTGTTCCAGCTCCATTTTC)	307	[34]
MH-MF1 (GCTTTCCCACGA ATAAATAATA) LepR (TAAACTTCTGGATGTCCAAAAAATCA)	407	[34]
MH-MF1 (GCTTTCCCACGA ATAAATAATA) HCO2198 (TAAACTT CAGGGTGACCAAAAAATCA)	407	[34]
LepF (ATTCAACCAATCATAAAGATATTGG) Ahl01R (RGGTATAACTATRAAAAAAATTAT)	145	[33]/this study
LCO1490 (GGTCAACAAATCATAAAGATATTGG) Ahl01R (RGGTATAACTATRAAAAAAATTAT)	145	[32]/this study
Ahl02F (ATTGGAGATGATCAAATTTATAAT) Ahl02R (TCAAAATCTYATATTATTTATTCG)	120	This study
Ahl03F (TTATAATTGGAGGATTTGGAAATTG) Ahl03R (AGTGGGGGGTAAACTGTTCATCC)	130	This study
Ahl04F (AGTAGAATTGTAGAAAATGG) Ahl04R (GTTGTAATAAAATTAATRGCTCC)	114	This study
Ahlfwd372F (GATCATCAGTTGATTTAGCTATT) Ahl05R (GTTAATARTATAGTAATAGCTCC)	168	This study
Ahl05F (ATTTTTTCTCTYCATTTAGCTGG) Ahl05R (GTTAATARTATAGTAATAGCTCC)	148	This study
Ahl06F (TATTTATTTGATCYGTAGGWATTAC) LepR (TAAACTTCTGGATGTCCAAAAAATCA)	133	This study/ [33]
Ahl06F (TATTTATTTGATCYGTAGGWATTAC) HCO2198 (TAAACTT CAGGGTGACCAAAAAATCA)	133	This study/ [32]

**Table 3:** Uncorrected pairwise genetic divergences (*p*-distances), %, with estimated standard errors (SEs); n/a, not available.

Taxon		1	2	3	4	5	6	7	8	9	10	11	12	13
1	*Callophrys* (*Ahlbergia*) *hmong* sp.n.	0.02 ± 0.02												
2	*Callophrys* (*Ahlbergia*) *tay* sp. n. (incl. *C*. (*A*.) *dao* sp. n.)	1.10 ± 0.39	0.07 ± 0.07											
3	*Callophrys* (*Ahlbergia*) *clarolinea*	0.60 ± 0.29	0.80 ± 0.33	0.00 ± 0.00										
4	*Callophrys* (*Ahlbergia*) *ferrea*	1.05 ± 0.38	0.96 ± 0.37	0.76 ± 0.34	0.00 ± 0.00									
5	*Callophrys* (*Ahlbergia*) *frivaldszkyi*	2.42 ± 0.58	1.87 ± 0.52	2.13 ± 0.55	1.98 ± 0.53	0.00 ± 0.00								
6	*Callophrys* (*Ahlbergia*) sp.	2.12 ± 0.55	1.56 ± 0.47	1.82 ± 0.51	1.67 ± 0.50	0.61 ± 0.29	0.00 ± 0.00							
7	*Callophrys* (*Cissatsuma*) *berezowskii*	2.19 ± 0.55	1.79 ± 0.49	1.90 ± 0.50	1.90 ± 0.51	0.53 ± 0.27	0.46 ± 0.24	0.15 ± 0.15						
8	*Callophrys* (*Cissatsuma*) *devyatkini* sp. n.	2.42 ± 0.58	1.87 ± 0.51	2.13 ± 0.54	1.98 ± 0.52	0.61 ± 0.30	0.61 ± 0.29	0.53 ± 0.26	0.00 ± 0.00					
9	*Callophrys* (*Novosatsuma*) *prodiga*	2.72 ± 0.64	2.17 ± 0.56	2.42 ± 0.59	2.28 ± 0.57	1.22 ± 0.45	0.91 ± 0.37	1.14 ±0.41	1.22 ± 0.43	n/a				
10	*Callophrys* (*Novosatsuma*) *collosa*	1.96 ± 0.53	1.41 ± 0.44	1.67 ± 0.48	1.52 ± 0.46	0.46 ± 0.26	0.15 ± 0.15	0.38 ± 0.21	0.46 ± 0.26	0.76 ± 0.34	n/a			
11	*Callophrys* (*Novosatsuma*) *magnapurpurea*	2.42 ± 0.59	1.87 ± 0.51	2.13 ± 0.54	1.98 ± 0.52	0.91 ± 0.38	0.61 ± 0.29	0.84 ± 0.34	0.91 ± 0.37	0.30 ± 0.22	0.46 ± 0.26	n/a		
12	*Callophrys* (*Callophrys*) *rubi*	2.12 ± 0.55	1.56 ± 0.47	1.82 ± 0.50	1.67 ± 0.49	0.61 ± 0.29	0.30 ± 0.20	0.53 ± 0.25	0.61 ± 0.30	0.91 ± 0.37	0.15 ± 0.15	0.61 ± 0.30	0.00 ± 0.00	
13	*Callophrys* (*Callophrys*) *avis*	1.66 ± 0.50	1.56 ± 0.49	1.37 ± 0.46	0.91 ± 0.36	2.13 ± 0.57	1.67 ± 0.51	1.90 ± 0.52	1.82 ± 0.52	2.43 ± 0.60	1.67 ± 0.51	2.13 ± 0.56	1.82 ± 0.53	0.00 ± 0.00

## Data Availability

All the analysed DNA sequences are available via the GenBank links provided (Table 1).
